# Supplementary figures and images for: Cognitive Capacity Limits Are Remediated by Practice-Induced Plasticity between the Putamen and Pre-Supplementary Motor Area
Source: eNeuro. 2020 Aug 27;7(4):ENEURO.0139-20.2020. doi: 10.1523/ENEURO.0139-20.2020 (PMC7458802; doi:10.1523/ENEURO.0139-20.2020)

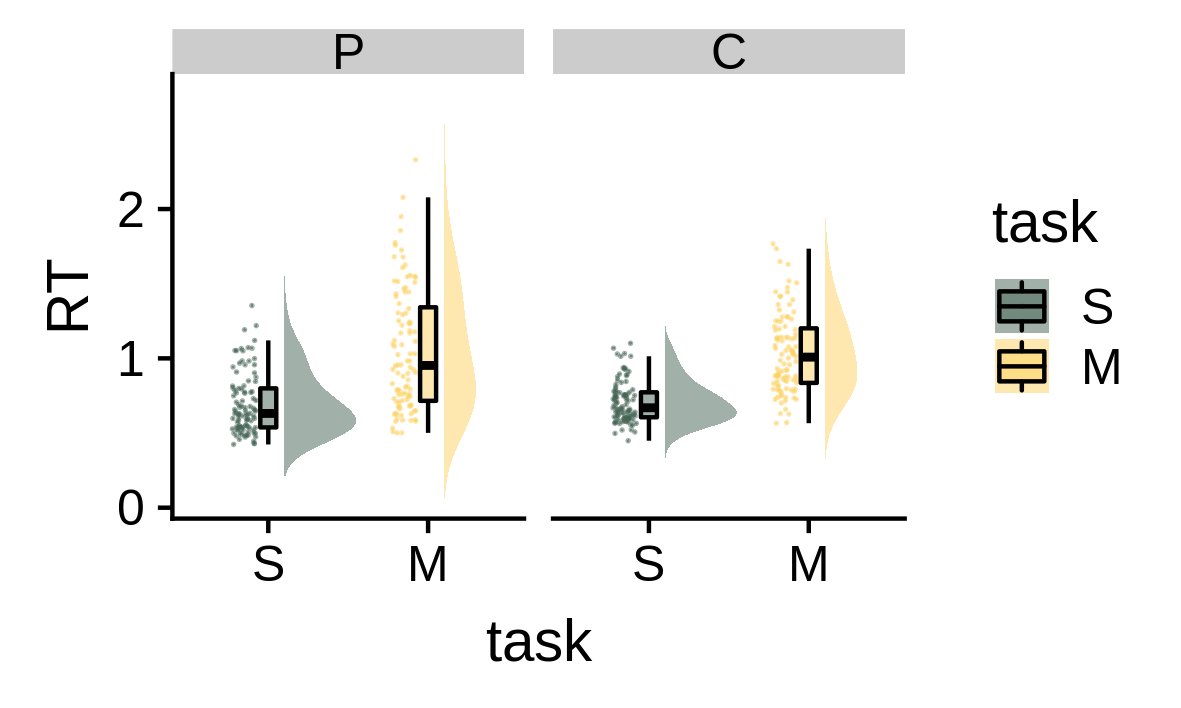

Supplement: Extended Data Figure 1-1 — Dot, box and density plots for mean RTs for the single-task (S) and multitask (M) for the practice and the control groups at the pretraining session. Download Figure 1-1, TIF file. [file enu-eN-NWR-0139-20-s01.tif]

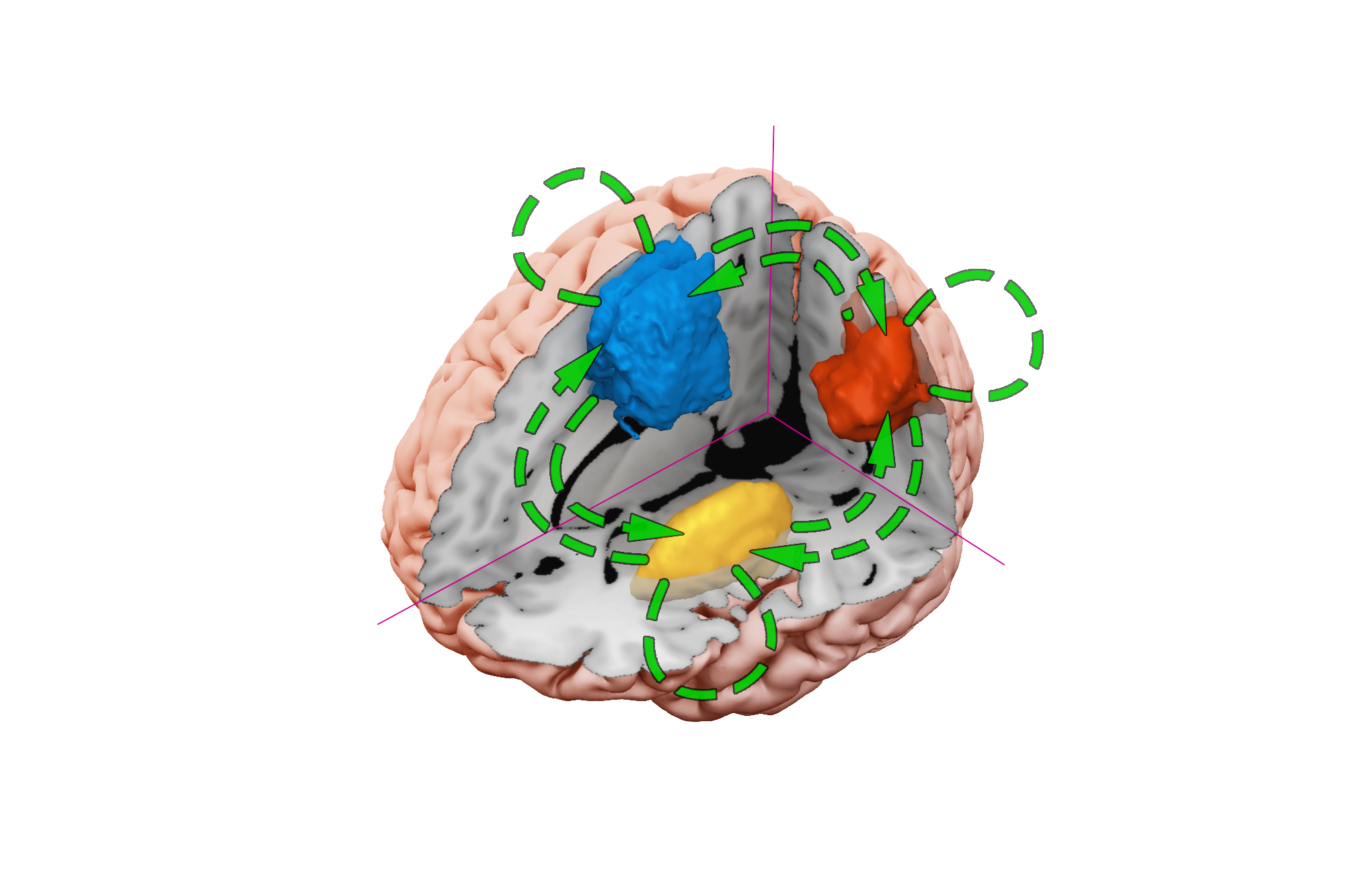

Supplement: Extended Data Figure 2-1 — The anatomical model (DCM.A) contained bidirectional endogenous connections between all three regions as well as endogenous self-connections. Download Figure 2-1, TIF file. [file enu-eN-NWR-0139-20-s02.tif]

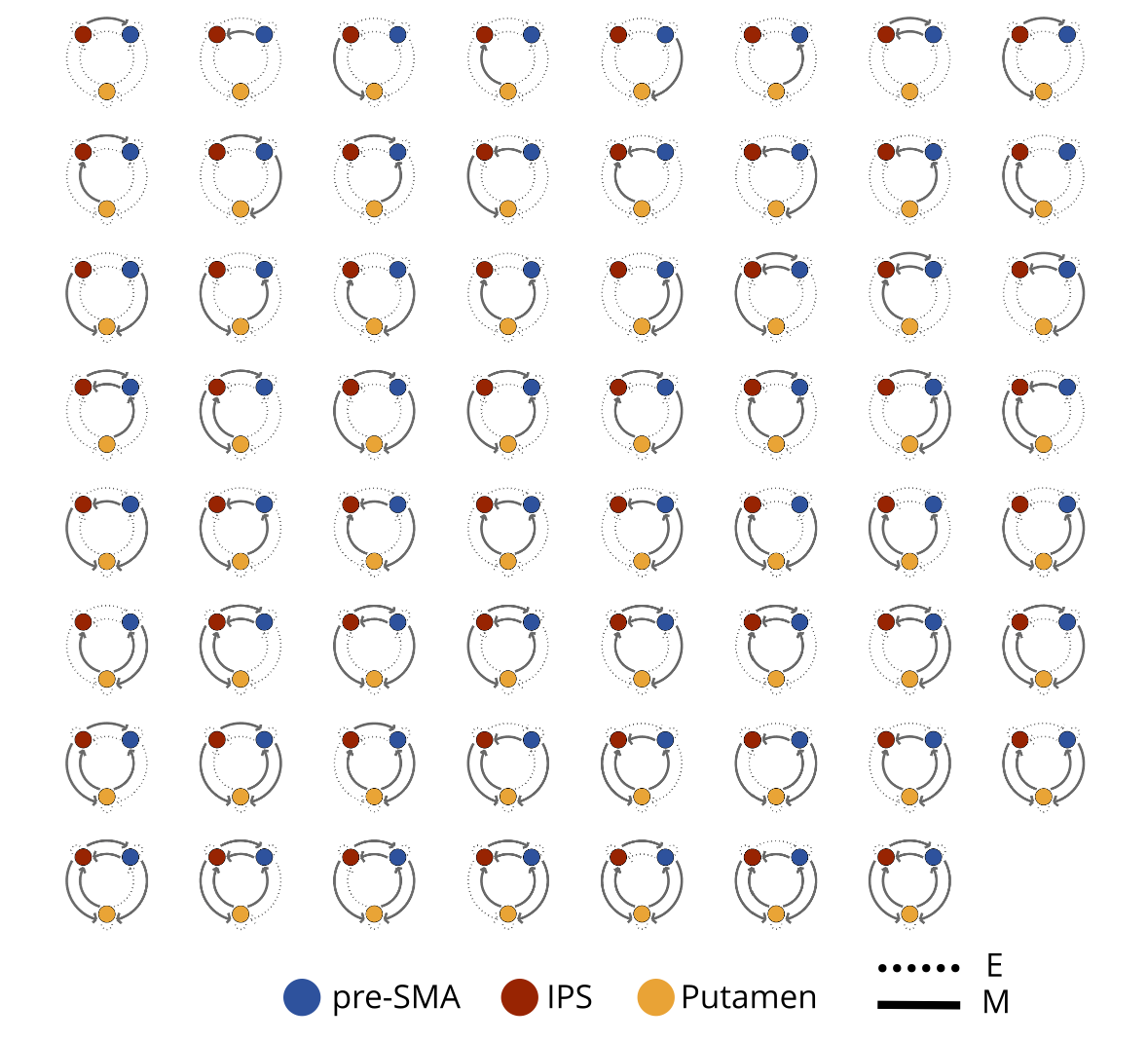

Supplement: Extended Data Figure 2-2 — We modelled all 63 possible modulatory influences of multitasking (DCM.B). E = endogenous connections, M = modulatory connections. Each pair of regions contains bidirectional coupling. Download Figure 2-2, TIF file. [file enu-eN-NWR-0139-20-s03.tif]

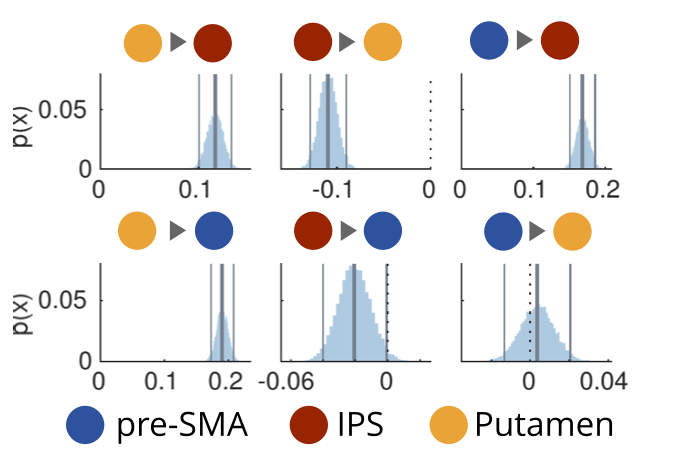

Supplement: Extended Data Figure 2-3 — Posterior probabilities over A parameters. p(x) = probability of sample from posterior density. Download Figure 2-3, TIF file. [file enu-eN-NWR-0139-20-s04.tif]

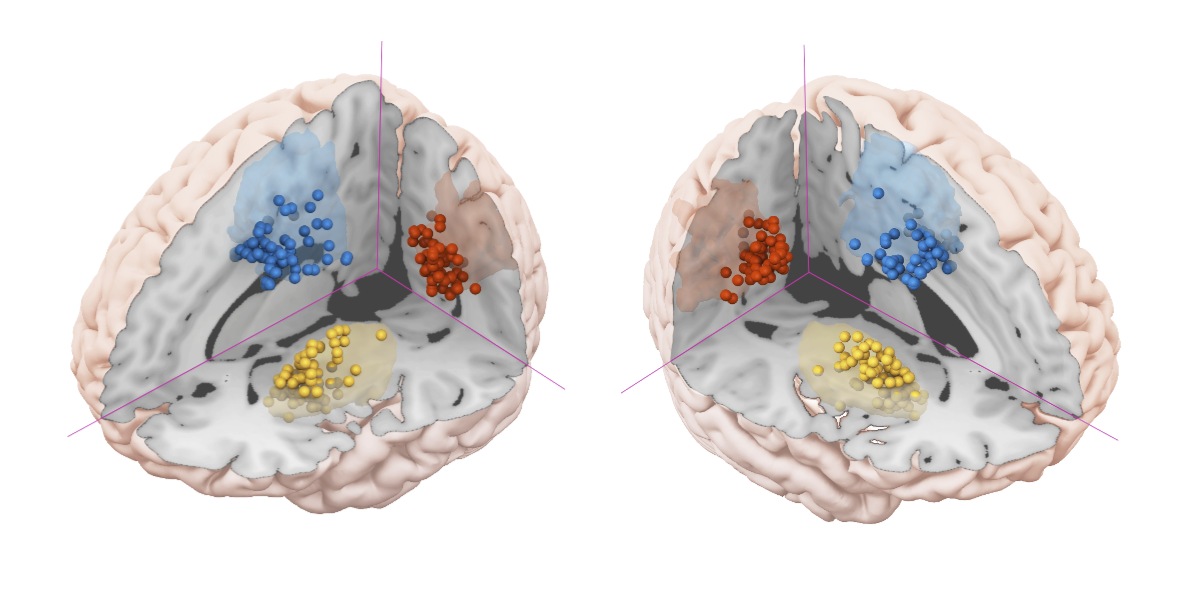

Supplement: Extended Data Figure 2-4 — Showing the individual peaks within each ROI for both the LH and RH data (1 sphere = 1 participant). Download Figure 2-1, TIF file. [file enu-eN-NWR-0139-20-s05.tif]

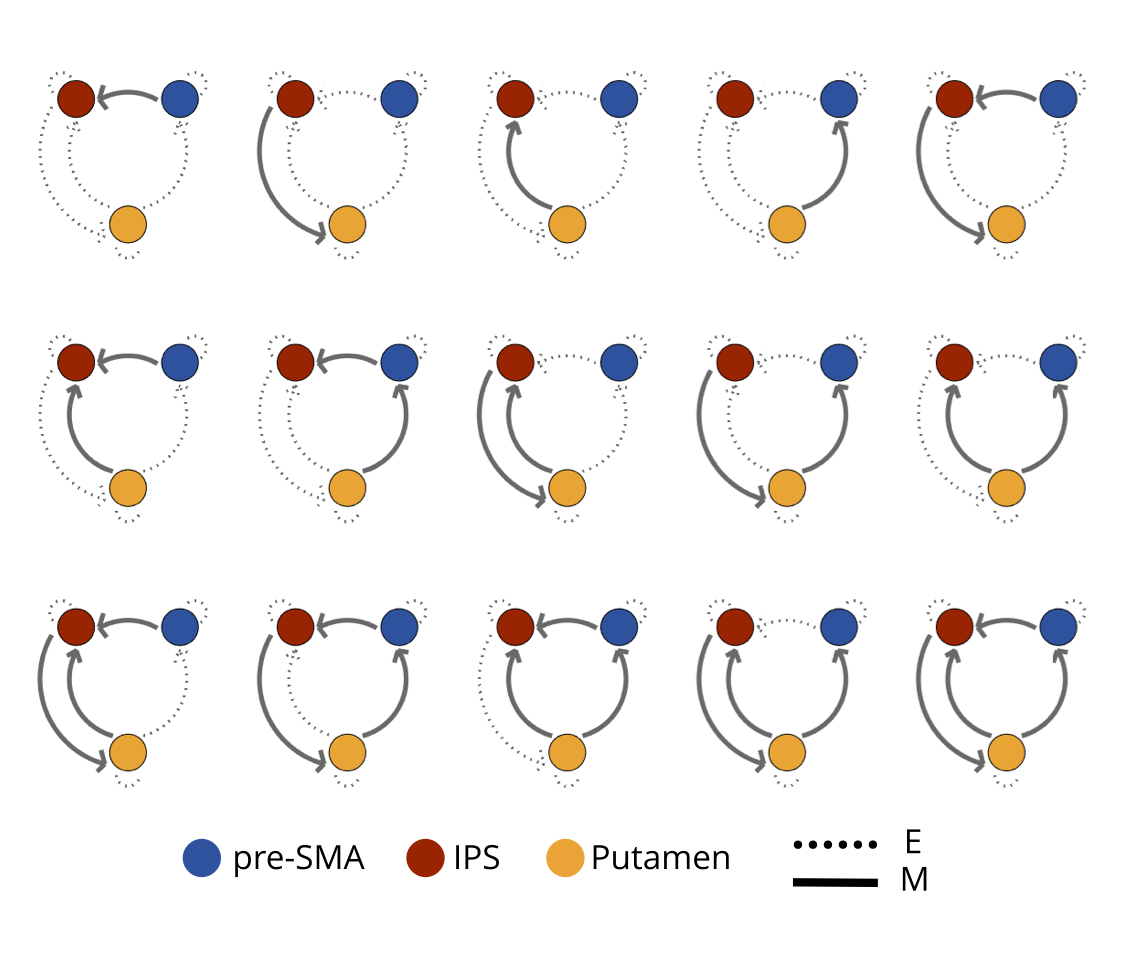

Supplement: Extended Data Figure 3-1 — Model space considered for the modulatory influence of practice (with models M = 1,…, 15). E = endogenous connections, M = modulatory connections. Download Figure 3-1, TIF file. [file enu-eN-NWR-0139-20-s06.tif]

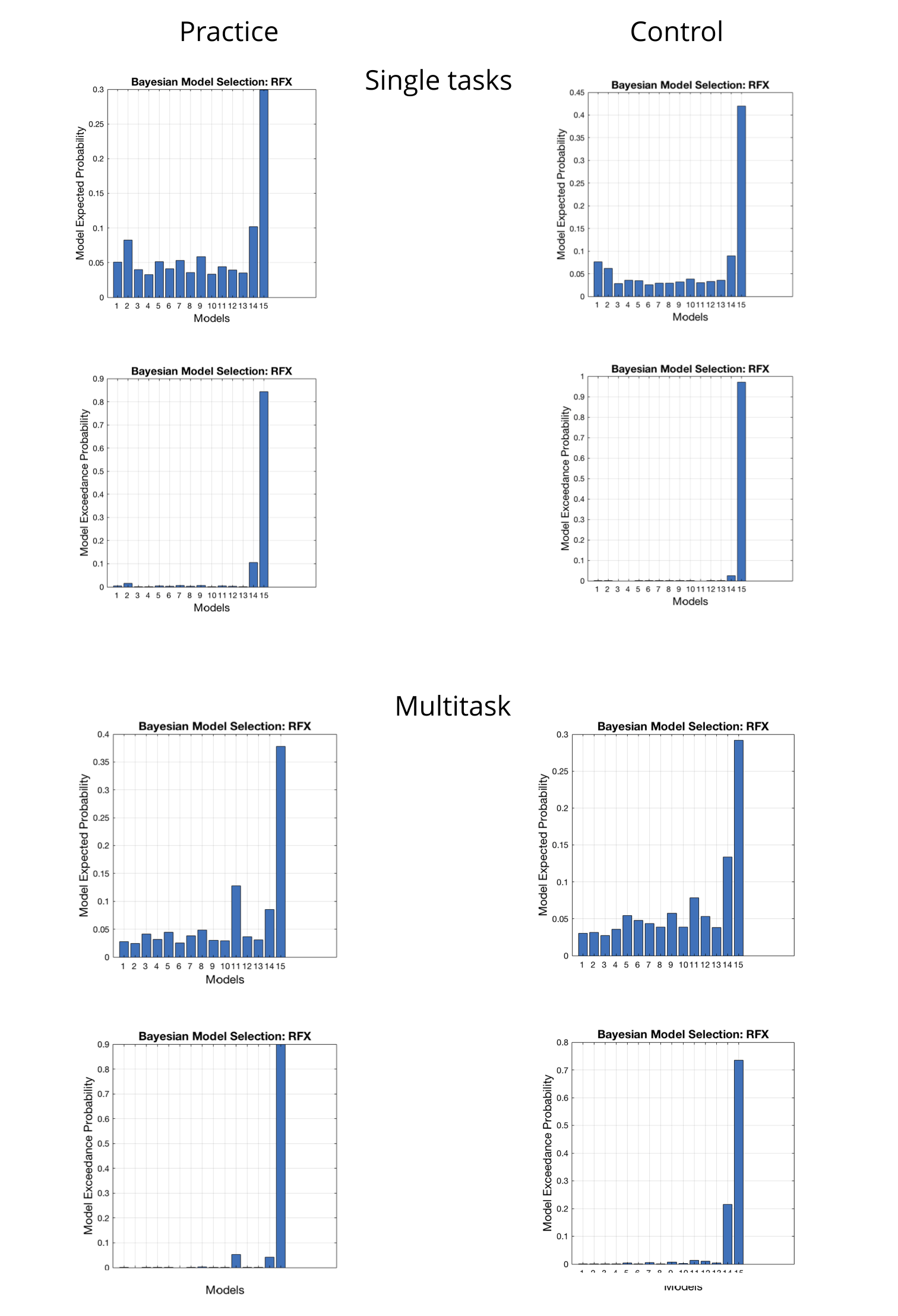

Supplement: Extended Data Figure 3-2 — Expected and exceedance model probabilities for single-task (top four panels) and for multitask (bottom four panels) data for the practice (left column) and control (right column) groups. Download Figure 3-2, TIF file. [file enu-eN-NWR-0139-20-s07.tif]

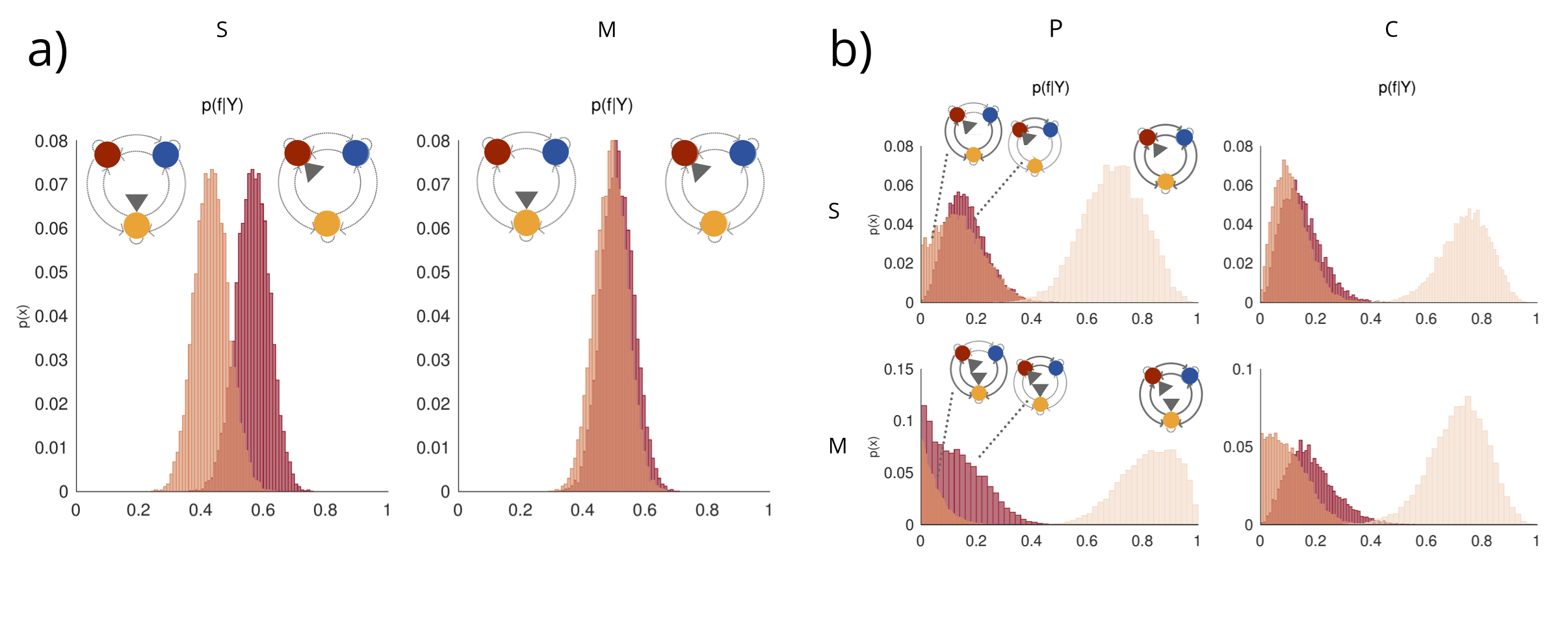

Supplement: Extended Data Figure 5-1 — Showing model family comparisons when modelling the modulatory influence of practice. A, Posterior probabilities over families, given the data [p(f|Y)], defined by inputs to IPS (left distribution) or putamen (right distribution) for single-task (S) or multitask (M) trials. B, Posterior probabilities over families differing in the connections modulated by multitasking (from left to right: corticostriatal modulations, corticocortical modulations, or both) for S and M trials, for the practice (P) and control (C) groups. Download Figure 5-1, TIF file. [file enu-eN-NWR-0139-20-s09.tif]
